# Influence of scan direction on subfoveal choroidal vascularity index using optical coherence tomography

**DOI:** 10.1038/s41598-022-20590-0

**Published:** 2022-10-05

**Authors:** Yung Hui Kim, Hyung Nam Jin, Hyun Jee Kim, Jong Hoon Lee, Yong-Sok Ji

**Affiliations:** Department of Ophthalmology and Research Institute of Medical Sciences, Chonnam National University Medical School and Hospital, 42 Jebong-ro, Dong-gu, Gwangju, 501-757 Korea

**Keywords:** Retina, Retina

## Abstract

We investigated the influence of scan direction on subfoveal choroidal vascularity index (CVI) measurements using spectral-domain optical coherence tomography (SD-OCT) in young healthy subjects. Seventy-eight eyes of 41 healthy volunteers were included. Choroidal structures were obtained using SD-OCT with enhanced depth imaging (EDI) through radial scans at the center of the macula. The subfoveal choroidal images in the horizontal (0°), 45°, vertical (90°) and − 45° directions were recorded and CVIs were analyzed according to their respective directions using image binarization. Additionally, subfoveal choroidal thickness (SFCT), and axial eye length were measured. The SFCT and subfoveal CVI showed a negative correlation but were only significant for the 45° scan (Pearson’s r = − 0.262, P = 0.021). The axial eye length and subfoveal CVI had no significant correlation in any direction (all P > 0.05). In the Bland–Altman plot, the subfoveal CVI measurement showed high agreement among the four scan directions. When the SFCT was ≥ 300 µm, there was no difference in the measured values of the subfoveal CVI among the four scan directions; however, when the SFCT was < 300 µm, there was a significant difference in subfoveal CVI among the scan directions (one-way analysis of variance, F = 4.685, P = 0.004). In subfoveal CVI measurement, it is considered that the horizontal (0°) scan can represent the vertical (90°) or oblique (45°, − 45°) scans. However, when the SFCT is thinner, the subfoveal CVI in each direction of radial scan may vary significantly. Hence, caution is required in the interpretation.

## Introduction

The choroidal vasculature lying between retina and sclera, has a key role of blood supply to the cone and rod photoreceptor cell layers. The choroid mainly consists of three layers which are choriocapillaris, Sattler’s and Haller’s layer^1^.

Due to the advent of detailed imaging techniques such as spectral-domain optical coherence tomography (SD-OCT) with enhanced depth imaging (EDI), it has recently become possible to analyze the specific choroidal layers^2^. With the higher resolution of OCT imaging, choroidal parameters such as choroidal thickness, choroidal vascularity index (CVI) have been widely studied in recent years. Several studies have shown that these choroidal parameters are influenced by some variables such as age, refractive error, axial eye length, ocular perfusion pressure, and intraocular pressure^3,4^.

CVI, defined by the proportion of the luminal area to the designated choroid area in OCT B-scan image, has been proposed as surrogate biomarker for monitoring various retinochoroidal diseases^5–12^. However, the analysis method is somewhat different for each study. Some authors used the CVI of the entire choroid in the central 30-degree B-scan images^6,8,10,12^, and others used the CVI of the smaller subfoveal area^5,7,9^. In addition, as mentioned above, it is known that CVI has an inter-individual variability and a limitation to clearly define the normal range. This limitation seems to be partly because the complex three-dimensional choroid structure is analyzed using two-dimensional plane images, and therefore some studies have attempted to investigate the choroid vasculature in three dimensions^13–16^.

Until recently, most studies on CVI used a single horizontal OCT B-scan image. Since the choroidal pachy-vessels, defined as dilated outer choroidal vessels^17–19^, tend to enter the posterior pole with oblique pattern from the equator, the authors hypothesized that CVI values would differ depending on the radial scan direction. To our best knowledge, however, there has been no study comparing CVI according to scan directions of OCT B-scans. Moreover, we do not know whether the CVI value, measured in only one directional OCT B-scan, can be an accurate indicator representing the three-dimensional choroidal structure.

The purpose of this study, therefore, was to evaluate the influence of scan direction on subfoveal CVI measurement using SD-OCT in young healthy subjects.

## Methods

This study was a cross sectional and observational study from December 2020 to July 2021 at the Department of Ophthalmology at Chonnam National University Hospital. Its conduct adhered to the tenets of the Declaration of Helsinki and was approved by the Institutional Review Boards of Chonnam National University Hospital (IRB No. BTMP-2021-352). Written informed consent was obtained from all subjects following an explanation of the purpose and study requirements.

### Subjects

Forty-one young healthy volunteers with a normal eye exam and a normal structural OCT of the macula were enrolled. Both eyes of each volunteer were examined. All eyes received detailed ocular examinations, which included assessment of refractive error using automated refractive keratometer (KR-8900, Topcon, Japan), best-corrected visual acuity, intraocular pressure, axial eye length by low-coherence interferometer (Lenstar LS900, Haag Streit AG, Koeniz, Switzerland), slit-lamp examination of the anterior segment and ophthalmoscopic fundus examination.

The inclusion criteria were as follows; age between 20 and 40 years; normal anterior segment by slit-lamp biomicroscopy; normal fundus and retinochoroid structure by ophthalmoscope and SD-OCT. The exclusion criteria were as follows; eyes with known ocular diseases such as diabetic retinopathy, uveitis, glaucoma and any chorioretinal diseases; presence of systemic diseases such as hypertension, dyslipidemia and diabetes; past history of intraocular surgery or intravitreal injections; media opacities such as cataract, vitreous opacity and vitreous hemorrhage.

### Optical coherence tomography imaging

**Figure 1:**
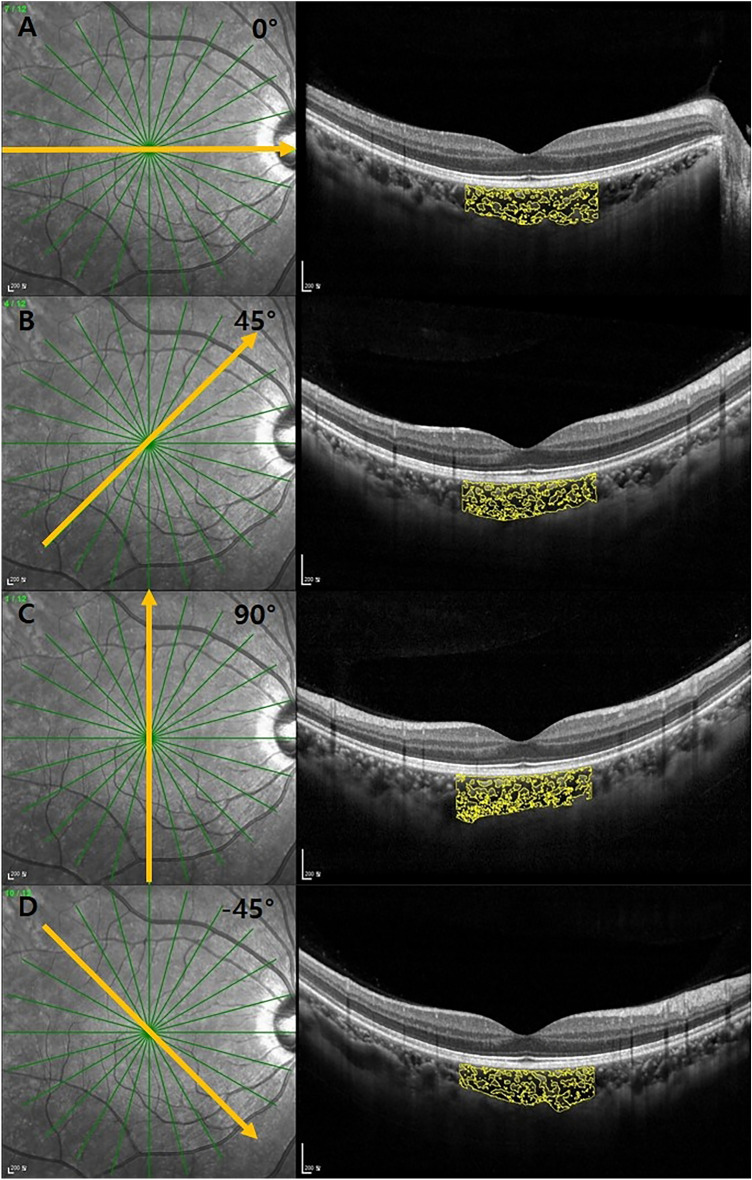
Overlay images of the region of interest (with a width of 3000 µm centered on fovea) on the EDI-OCT scan according to radial scan direction. Horizontal (0°) (**A**), oblique (45°) (**B**), vertical (90°) (**C**) and oblique (− 45°) (**D**) B-scan images. *EDI* extended-depth image, *OCT* optical coherence tomography.

EDI-OCT images using Spectralis OCT device (Spectralis; Heidelberg Engineering; Heidelberg, Germany) were obtained from healthy volunteers without pupillary dilation by the same experienced examiner and reviewed individually by two investigators (Y.H.K. and Y.S.J.) for image quality evaluation. EDI-OCT protocols are as follows; The OCT device was positioned in close proximity to the volunteer’s eye in order to acquire an inverted image; 12 equally-angled radial OCT B-scan sections were obtained in 30-degree width centered on the fovea; Each radial section had 100 OCT frames averaged with automatic real time mode. Individual OCT B-scan had a horizontal length of 768 pixels. Only ≥ 15 of signal strength of the OCT images were included in the study. Therefore, 4 eyes with a signal strength of < 15 were excluded, and a total of 78 eyes of 41 healthy volunteers were finally included in this study. Among these 12 radial scans, four OCT B-scans of horizontal (0°), vertical (90°), and oblique (45° and − 45°) directions were used for image binarization and subfoveal choroidal structure analysis. The oblique direction of a 45° is from inferior-temporal to superior-nasal, and − 45° is from superior-temporal to inferior-nasal (Fig. 1).

### Image binarization and measurement of subfoveal choroid structures

The binarization of the subfoveal choroidal area was performed by one of the authors (Y.H.K.) using a public domain software, Fiji (http://imageJ.net/software/fiji)^20^. The binarization protocol described by Sonoda et al.^21^ with minor adjustments was used in this study. After mounting the OCT B-scan images on Fiji, the scale setting was done by converting pixels to microns (In the Fiji program, 200 μm was equivalent to 16 pixels that was same as a scaling size of 12.5 μm/pixel in the SD-OCT B-scan image.) using the horizontal scale present on left lower side of SD-OCT B scan images, so that the subfoveal choroidal region with a width of 3000 µm (1500 µm on either side of fovea center) could be drawn manually by the polygon tool. The upper border of the region of interest was traced along the choroidal-retinal pigment epithelium junction and the lower border along the choroidal-scleral junction. This choroidal area was added to Region of Interest Manager Tool. The images were converted to 8-bit images to allow the application of Auto Local Threshold Tool. Among various types of Auto Local Threshold Tools, Niblack’s thresholding method was known to have substantially high resolution and demarcation of vascular and stromal area^21^. Hence, Niblack Auto Local Threshold was utilized in all images in this study. In the Fiji program, the radius value was set as 15. White object on black background was selected to white pixels with values above the threshold value. Special parameters 1 and 2 of Niblack’s thresholding method were set as default. The default value of parameter 1 was 0.2 for bright objects and − 0.2 for dark objects. The default value of parameter 2 was 0. The subfoveal CVI, defined as the vascular area divided by the total choroidal area in the selected region of interest, was then calculated through the “analyze particles”. Figure 1 shows the overlay images of the region of interest on the EDI-OCT scan according to radial scan direction. In addition, subfoveal choroidal thickness (SFCT) was measured at central fovea using calipers built in the Fiji software from the choroidal-retinal pigment epithelium junction to the choroidal-scleral junction. The mean value of the SFCT from each of the 4 radial scans was used for analysis.

### Statistical analysis

Statistical analysis was performed using Statistical Package for Social Sciences version 22.0 for Windows (SPSS Inc., Chicago, IL, USA) and GraphPad Prism version 7.0 (GraphPad Software, La Jolla, CA, USA). The normal distribution for all variables was assessed using the Kolmogorov–Smirnov test. Data are presented as the mean ± standard deviation (SD). Differences among groups for continuous variables were assessed using the one-way analysis of variance (ANOVA). Differences between groups for categorical variables were assessed using the Chi-square test. The correlation between the subfoveal CVI and the axial eye length and SFCT were assessed by Pearson correlation analysis. The agreements of the subfoveal CVI measurement among different radial scan directions were analyzed by Bland–Altman plots. Intra-class correlation coefficient (ICC) value of 0.75–1.00 indicates good agreement. A *P*-value of less than 0.05 was considered to have statistical significance.

## Results

**Figure 2:**
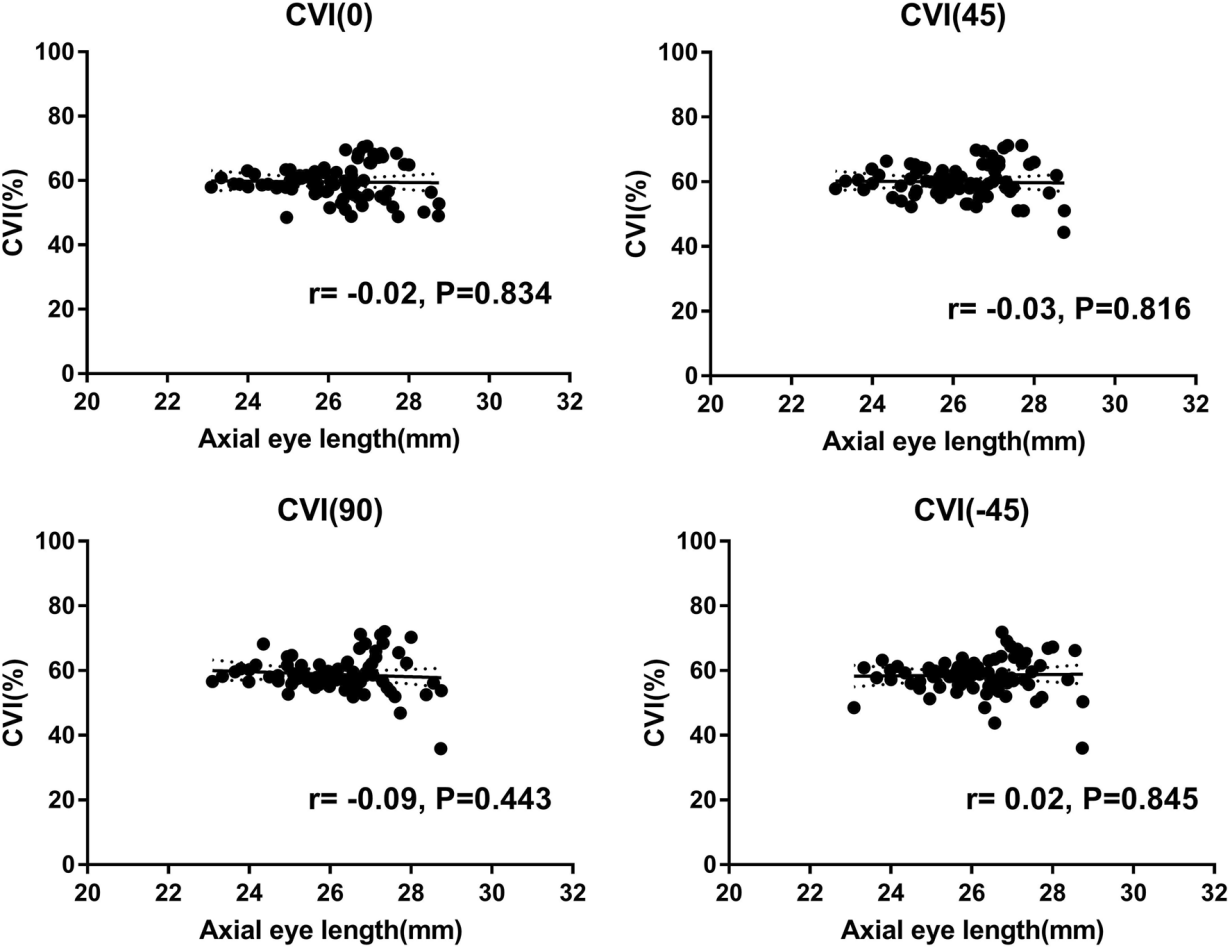
Correlation analysis between the axial eye length and the subfoveal CVI. There were no significant correlations in all radial scan directions (all p > 0.05). CVI = choroidal vascularity index.

**Figure 3:**
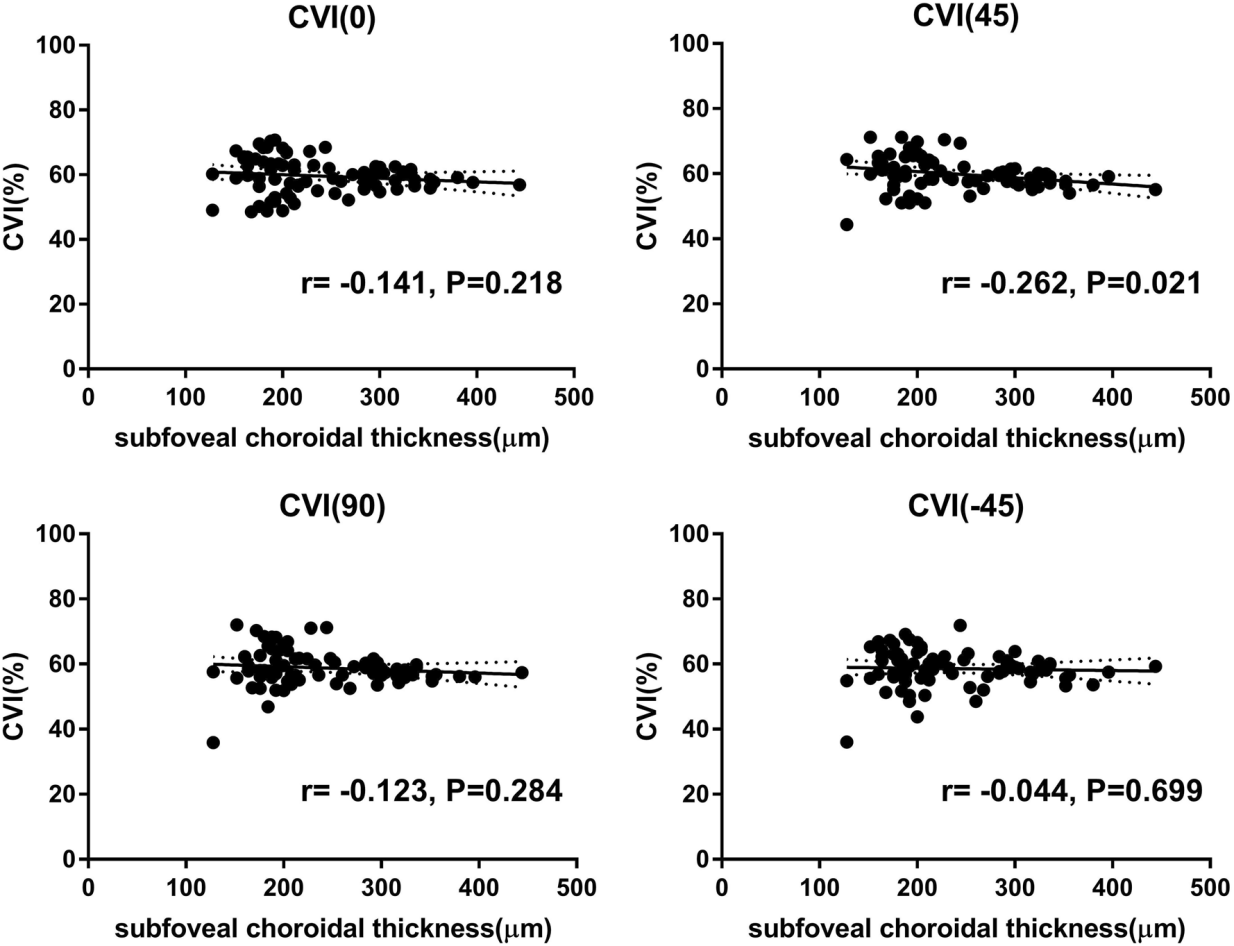
Correlation analysis between the subfoveal choroidal thickness (SFCT) and the subfoveal CVI. There were no significant correlations in all scan directions other than oblique 45° scan direction. The SFCT and the subfoveal CVI in 45° scan direction showed a negative correlation (Pearson's r = − 0.262, p = 0.021). *CVI* choroidal vascularity index.

All eyes had the best-corrected visual acuity of 20/20. Mean age of participants was 25.26 ± 2.41 years. In terms of choroidal characteristics, mean SFCT was 239.95 ± 68.96 µm and mean subfoveal CVI of all scan directions was 59.24 ± 4.90%. There were no differences among the subfoveal CVIs according to radial scan direction (P > 0.05) (Table 1). In the correlation analysis between the axial eye length and the subfoveal CVI, no significant correlation showed in all radial scan directions (axial eye length vs. subfoveal CVI(0°), Pearson’s r = − 0.02, P = 0.834; axial eye length vs. subfoveal CVI(45°), Pearson’s r = − 0.03, P = 0.816; axial eye length vs. subfoveal CVI(90°), Pearson’s r = − 0.09, P = 0.443; axial eye length vs. subfoveal CVI(− 45°), Pearson’s r = 0.02, P = 0.845) (Fig. 2). The SFCT and the subfoveal CVI in 45° had a negative correlation (Pearson’s r = − 0.262, P = 0.021). There were no significant correlations between the SFCT and the subfoveal CVI in horizontal (0°), vertical (90°), and − 45°, respectively (all P > 0.05) (Fig. 3).

**Table 1:** Demographic characteristics and choroidal parameters of SD-OCT in healthy subjects.

	Value
Age (years)	25.26 ± 2.41
Sex (M/F)	20/21
Axial eye length (mm)	25.98 ± 1.26
SFCT (µm)	239.95 ± 68.96
**Subfoveal CVI (%)**	
0°	59.58 ± 5.43
45°	59.91 ± 5.06
90°	58.83 ± 5.55
− 45°	58.59 ± 5.61
Average subfoveal CVI	59.24 ± 4.90

All continuous variables were presented as mean ± standard deviation.

*SD-OCT* spectral-domain optical coherence tomography, *SFCT* subfoveal choroidal thickness, *CVI* choroidal vascularity index.

**Figure 4:**
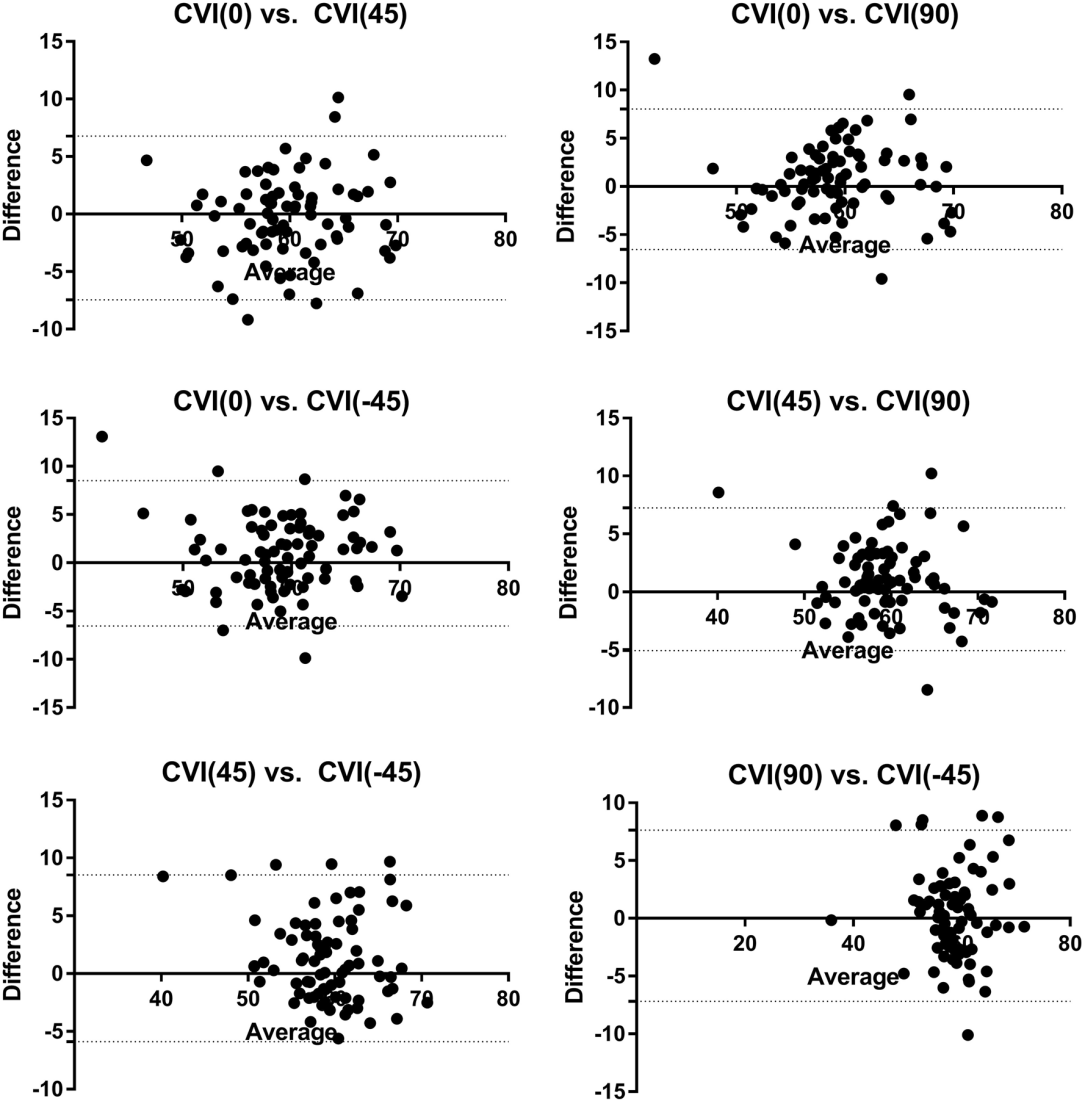
Bland–Altman plot showing the agreements between the subfoveal CVI in the different radial scan direction. All the measurements of the subfoveal CVI in the different radial scan direction showed high agreements. (0° vs. 45°, intraclass correlation coefficient (ICC) = 0.76, 95% limit of agreement (LoA) − 7.44 to 6.78; 0° versus 90°, ICC = 0.77, 95% LoA − 6.53 to 8.04; 0° versus − 45°, ICC = 0.76, 95% LoA − 6.53 to 8.50; 45° versus 90°, ICC = 0.83, 95% LoA − 5.06 to 7.24; 45° versus − 45°, ICC = 0.77, 95% LoA − 5.90 to 8.53; 90° versus − 45°, ICC = 0.77, 95% LoA − 7.17 to 7.63).

In the Bland–Altman plot, all measurements of the subfoveal CVI in the different radial scan directions showed respective high agreements (0° vs. 45°, intraclass correlation coefficient [ICC] = 0.76, 95% limit of agreement [LoA] − 7.44 to 6.78; 0° vs. 90°, ICC = 0.77, 95% LoA − 6.53 to 8.04; 0° vs. − 45°, ICC = 0.76, 95% LoA − 6.53 to 8.50; 45° vs. 90°, ICC = 0.83, 95% LoA − 5.06 to 7.24; 45° vs. − 45°, ICC = 0.77, 95% LoA − 5.90 to 8.53; 90° vs. − 45°, ICC = 0.77, 95% LoA − 7.17 to 7.63; Fig. 4).

**Figure 5:**
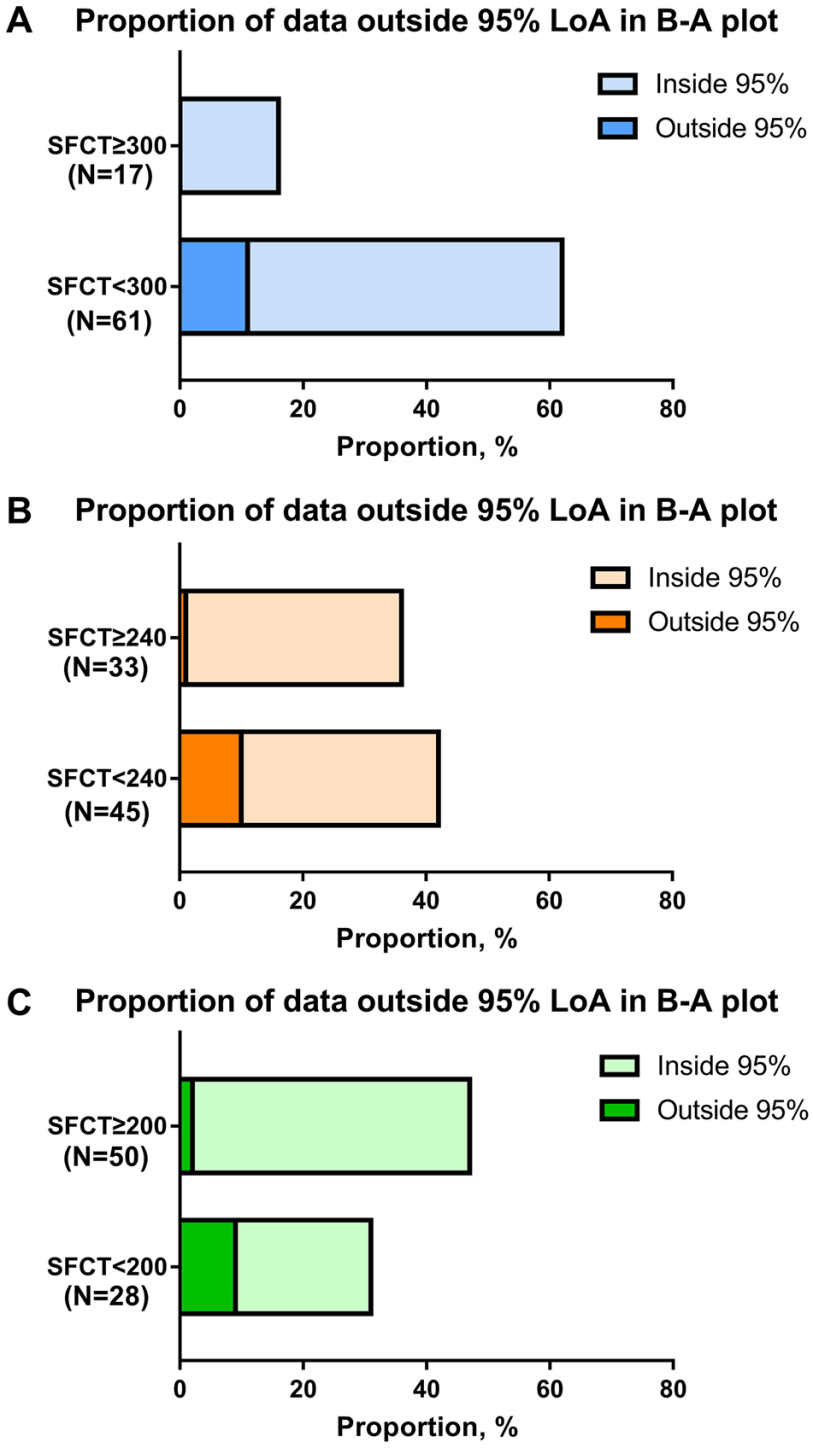
The distribution of data outside the 95% limit of agreement (LoA) in the Bland–Altman plot was analyzed according to the subfoveal choroidal thickness (SFCT). Data outside 95% LoA in the Bland–Altman plot had a larger proportion in the group of SFCT with < 300 µm (17.74%), compared to the group of SFCT with ≥ 300 µm (0.00%) (**A**). Data outside 95% LoA in the Bland–Altman plot had a larger proportion in the group of SFCT with < 240 µm (22.22%), compared to the group of SFCT with ≥ 240 µm (3.03%) (OR = 9.143; 95% CI [1.35–102.1]) (**B**). In addition, similar result was shown in the group of SFCT with < 200 µm (29.03%), compared to the group of SFCT with ≥ 200 µm (4.26%) (OR = 9.205, 95% CI [2.004–44.05]) (**C**).

The distribution of data outside the 95% LoA was analyzed according to SFCT. More data from the < 300 µm SFCT group (N = 61) fell outside the 95% LoA in the Bland–Altman plot compared to that of the ≥ 300 µm group (N = 17) (17.74% vs. 0.00%; Fig. 5A). Similarly, more data from the < 240 µm SFCT group (N = 45) fell outside the 95% LoA in the Bland–Altman plot compared to that of the ≥ 240 µm SFCT group (N = 33) (odds ratio [OR] = 9.143; 95% confidence interval [CI] [1.35 to 102.1], 22.22% vs. 3.03%, P < 0.05; Fig. 5B). It was based on the average SFCT value of 240 µm (Table 1). Again, similar results were found in the group of SFCT with < 200 µm (N = 28), compared to the group of SFCT with ≥ 200 µm (N = 50) (OR = 9.205; 95% CI [2.004 to 44.05], 29.03% vs. 4.26%, P < 0.01; Fig. 5C).

**Figure 6:**
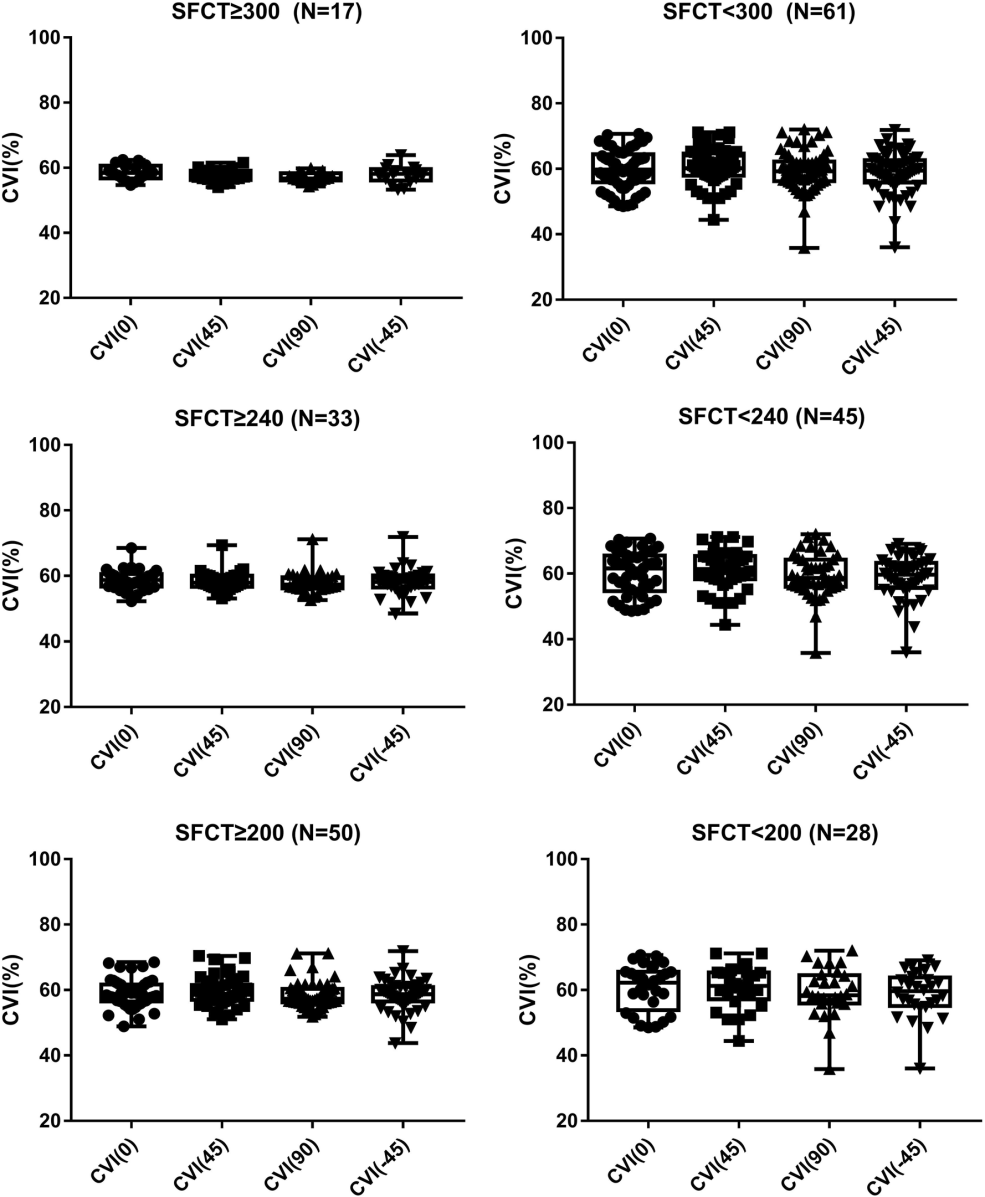
The difference of subfoveal CVI among the radial scan directions depending on the SFCT. When the SFCT < 300 µm, there was a statistically significant difference in subfoveal CVI among the scan directions (P = 0.004). When the SFCT < 240 µm, there was a statistically significant difference in subfoveal CVI among the scan directions (P = 0.004). In addition, When the SFCT < 200 µm, there was a statistically significant difference in subfoveal CVI among the scan directions (P = 0.018). There were no significant differences of subfoveal CVI among the scan directions in the group of SFCT ≥ 300 µm, SFCT ≥ 240 µm, and SFCT ≥ 200 µm (all P > 0.05). *CVI* choroidal vascularity index, *SFCT* subfoveal choroidal thickness.

When the SFCT was < 300 µm (N = 61), there was a statistically significant difference in subfoveal CVI among the scan directions (one-way ANOVA, F = 4.69, P = 0.004). When the SFCT was < 240 µm (N = 45), there was a significant difference in subfoveal CVI among the scan directions (one-way ANOVA, F = 4.72, P = 0.004). Additionally, when the SFCT was < 200 µm (N = 28), there was a significant difference in subfoveal CVI among the scan directions (one-way ANOVA, F = 5.81, P = 0.018). There were no significant differences of subfoveal CVI among the scan directions in the group of SFCT ≥ 300 µm (N = 17) (one-way ANOVA, F = 2.74, P = 0.07), SFCT ≥ 240 µm (N = 33) (one-way ANOVA, F = 1.16, P = 0.326), and SFCT ≥ 200 µm (N = 50) (one-way ANOVA, F = 2.30, P = 0.09), respectively (Fig. 6).

## Discussion

The choroid is a densely vascularized structure that contributes to the huge amount of oxygen and nutrients supply to the outer sensory retina and retinal pigment epithelium. Over the last decades, OCT with EDI mode has allowed detailed and non-invasive analysis of chorioretinal structures. In particular, the CVI is emerging as a useful indicator to analyze the choroidal structure in the eyes of various chorioretinal diseases, discriminating and quantifying the vascular and stromal components. It is known that the CVI varies depending on some factors including age and intraocular pressure^22–24^. In addition to these factors affecting the CVI measurements, authors aimed to investigate the effect of the scan direction of OCT on the CVI measurements. To our best knowledge, this is the first study to analyze the difference of the subfoveal CVI according to the radial scan direction of SD-OCT.

The present study showed no significant differences in the mean value of subfoveal CVI of all healthy subjects in reference to radial scan direction. This finding may be due to the offset effect of averaging the CVI values for each scan direction. Additionally, no significant correlation between axial eye length and subfoveal CVI for each radial scan direction was found in the present study. A similar result was found in the correlation analysis between the SFCT and the subfoveal CVI for each radial scan direction. In particular, the SFCT showed a significantly negative correlation with the subfoveal CVI in only the 45° scan direction. In the study conducted by Singh^23^, CVI using horizontal scan showed a wide topographic variation in which the highest CVI was noted in the nasal area, followed by the inferior, temporal, and superior quadrants, with the lowest CVI in the subfoveal area. Shin et al.^25^ also investigated that a three-dimensional topographic map of choroidal thickness in Early Treatment Diabetic Retinopathy Study subfields and demonstrated the gradual nasal thinning of the choroid. However, since correlation coefficient values of the subfoveal CVI with the SFCT of each scan direction are quite low in this study, caution is needed in interpreting the result.

In contrast to the current study, Agrawal et al.^26^ showed that a thicker SFCT was significantly associated with a higher CVI. The differences in these results are possibly due to the cohort of the Agrawal’s study being 45 to 85 years of age, whereas this study was conducted with a younger cohort, the average age of which was 25 years. It can be hypothesized that the choroidal luminal area with CVI decreases with ageing, whereas the choroidal stromal area remains stable. That is, in the case of young healthy eyes, the subfoveal CVI can be considered to remain within the normal range even if there is a difference in the SFCT or axial eye length.

Previous studies have shown that CVI measurement using horizontal scans of the subfovea is representative of that of the posterior pole^5,7,26–28^. In this study, there was generally high agreement between radial scan directions in the Bland–Altman plot. The 95% LoA ranged from a minimum of − 7.44% to a maximum of + 8.53%. The ICCs showed a relatively high degree of agreement with 0.76 or more, suggesting that measuring the subfoveal CVI using only a horizontal (0°) scan can be representative of using a vertical (90°) or oblique (45°, − 45°) scan direction.

**Figure 7:**
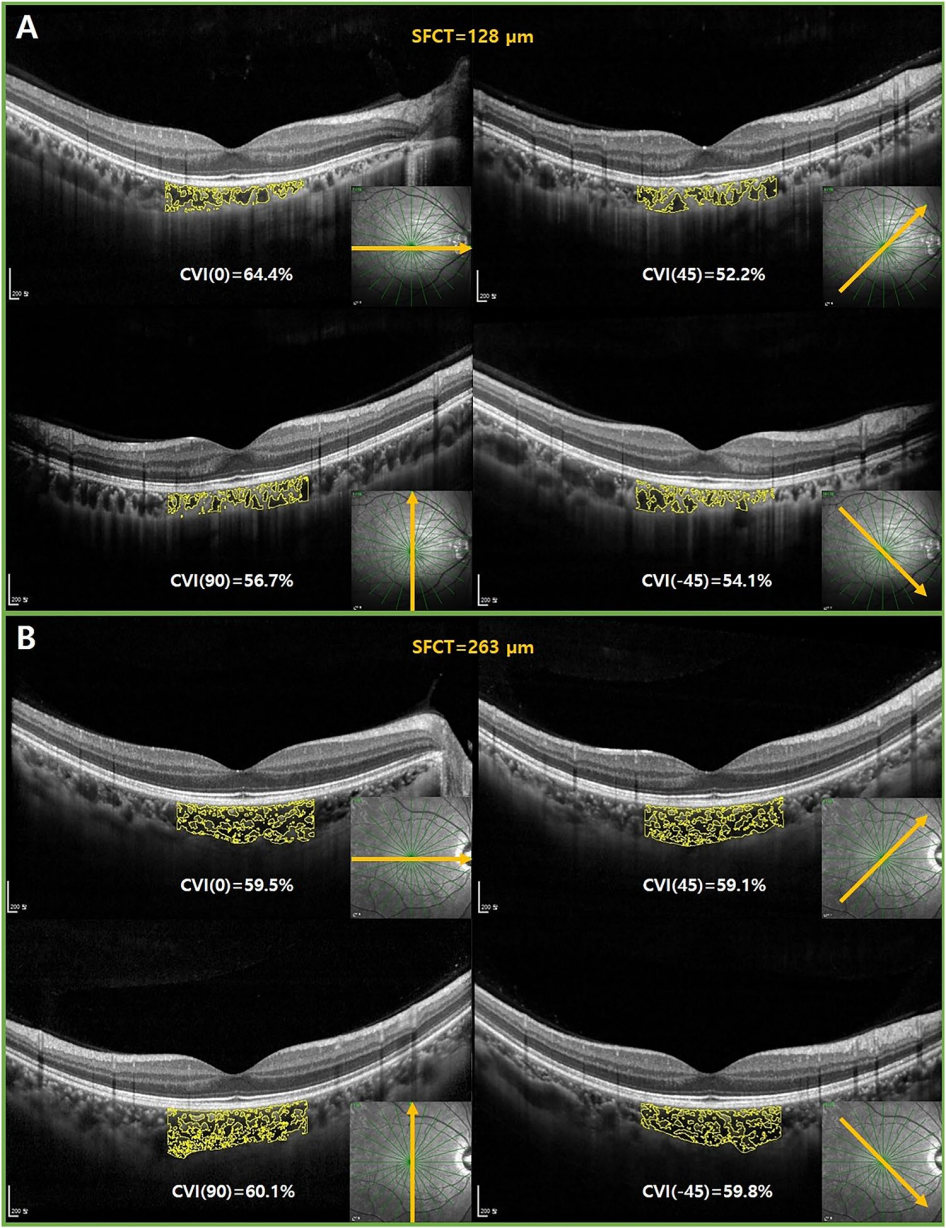
It shows examples that how different the subfoveal CVI is among radial scan directions depending on the SFCT. The subject with thin SFCT (128 µm) showed large variation of subfoveal CVI among radial scan directions (**A**). The subject with normal range of SFCT (263 µm) showed little variation of subfoveal CVI among radial scan directions (**B**). *CVI* choroidal vascularity index, *SFCT* subfoveal choroidal thickness.

Interestingly, a thinner SFCT was correlated with a larger difference in subfoveal CVI among scan directions in this study. Figure 7A shows an example of eye with thin choroid in which the subfoveal CVI is significantly different for each scan direction. However, Fig. 7B exhibits little variation of subfoveal CVI among radial scan directions in eyes with normal range of SFCT. Alshareef et al.^29^ found that the choroidal stroma was smaller in myopes, while the choroidal luminal area showed no difference between non-myopes and myopes. Gupta et al.^3^ also suggested that a pathogenesis of myopic choroidal thinning may involve both choroidal vascular and choroidal stroma. Rather than choriocapillaris, large choroidal vessels like Haller’s or Sattler’s layer largely affect the measurement of the CVI. Therefore, eyes with thinner choroid may show a significant difference depending on the running pattern of the choroidal vasculature.

Our study has a few limitations. First, only young healthy individuals were included in this study. Although horizontal scan of subfoveal CVI represents the CVI of entire macula area in normal eye, it may not be applicable in patients with localized chorioretinal disease. Second, authors investigated both eyes of each participant, not considering inter-eye differences which should be small. Third, there is a potential error of the image binarizing method, resulting in over- or underestimation of choroidal stroma and luminal area. Fourth, we analyzed only a small area with a radius of 1500 μm centered on the fovea, not an entire fundus. Previous studies^30,31^ showed that the measurements accuracy of the real scale of fundus images rely on Littman’s curves with respect to the axial eye length. However, even without considering Littman’s method, since the CVI value is a ratio, the results might not be significantly affected in this study. Fifth, only four scans out of 12 radial scan directions were used for analysis. Using all 12 scan directions would provide more information. The authors considered, however, maximizing each radial angle difference by 45° can reveal the difference in subfoveal CVI more clearly among radial scan directions. Lastly, since the subfoveal choroidal region for analysis was drawn manually by one grader, inter-observer variability could not be evaluated.

Despite these limitations, our study demonstrated that young healthy eyes maintain the normal range of subfoveal CVI regardless of choroidal thickness. Additionally, it is noteworthy that even in healthy eyes, there can be a difference in subfoveal CVI based on radial scan direction depending on the choroidal thickness.

In conclusion, it is considered that the subfoveal CVI measurement using a single horizontal OCT scan can represent the overall CVI. In the case of thin SFCT, however, the subfoveal CVI in each direction of radial scan may vary significantly, so caution is required in the interpretation.

## Data Availability

The datasets generated during the current study are available from the corresponding author on reasonable request.
